# The relationship between gender, parenthood and practice intentions among family medicine residents: cross-sectional analysis of national Canadian survey data

**DOI:** 10.1186/s12960-019-0396-y

**Published:** 2019-08-15

**Authors:** Miriam Ruth Lavergne, Andrea Gonzalez, Megan Alyssa Ahuja, Lindsay Hedden, Rita McCracken

**Affiliations:** 1 Faculty of Health Sciences, Simon Fraser University, 8888 University Drive, Blusson Hall 10502, Burnaby, BC V5A 1S6 Canada; 2 Department of Family Medicine, University of British Columbia, 5950 University Boulevard, Vancouver, BC V6T 1Z3 Canada; 3 Centre for Health Services and Policy Research, University of British Columbia, 2206 East Mall, Vancouver, BC V6T 1Z3 Canada

**Keywords:** Health human resources, Family medicine, Residents, Practice intentions, Parenthood, Gender, Workforce planning

## Abstract

**Background:**

Family medicine (FM) residents choose among a range of options as they enter practice, including practice model, clinical domains, settings, and populations. The choices they make have implications for primary care workforce planning and may differ between FM residents who are parents and those who are not, as well as between male and female FM residents. We investigate whether parenthood shapes intentions among FM residents entering practice and whether the effect of parenthood differs between male and female FM residents.

**Methods:**

We conducted cross-sectional analysis of national survey data collected from FM residents in Canadian residency programs by the College of Family Physicians of Canada between 2014 and 2017. The survey captures information on intentions for comprehensive or focused practice, practice model, clinical domains, practice setting, and populations. We used chi-square tests and multivariable logistic regression to investigate the relationships between parenthood, gender, and practice intentions, adjusting for other physician personal characteristics.

**Results:**

Almost a quarter of FM residents were parents or became parents during residency. Intentions for the provision comprehensive care were higher among parents, and intentions for clinically focused practice were lower. Differences in intentions for practice models, domains, and settings/population were primarily by gender, though in several cases the effects of parenthood differed between female and male FM residents. Even during residency, the effects of parenthood differ between male and female residents: while three quarters of male parents finish residency in two years, fewer than half of female parents do.

**Conclusions:**

Both parenthood and gender independently shape practice intentions, but the effect of parenthood differs for male and female FM residents. Supporting FM residents who are parents may positively impact the quality and availability of primary care services, especially since parents are more likely to report intentions to provide  comprehensive care soon after entering practice.

## Background

Primary care is the first and main access point into the health care system in Canada. While the number of primary care physicians per capita continues to rise in Canada [1], almost 15% of Canadians still report not having a regular care provider [2]. One reason for this discrepancy may be that not all family medicine (FM) graduates end up going on to a comprehensive (generalist) practice [3, 4]. FM residents have a wide range of practice options available to them in Canada. Some become hospitalists or emergency doctors, some work at walk-in clinics, some provide specialized care in long-term care facilities or palliation, some adopt other forms of focused practice, and some choose a combination of these practice options. A better understanding of what factors shape practice intentions can inform health workforce planning, and policies that support doctors in their transition from residency to practice, toward the goal of ensuring all Canadians have access to primary care.

Much of the literature on differences in physician practice patterns focuses on the effect of gender [5]. In industrialized countries, the proportion of female primary care physicians has almost doubled in the last 30 years [6]. In Canada, the number of female medical students surpassed male students in 1995/1996 [7]. Among practicing physicians in 2017, 45.5% of family medicine physicians were female compared with 36.2% of specialist physicians [1]. Data from the Canadian Residency Matching Service (CaRMS) shows that more women than men continue to choose FM as their preferred residency training [8].

Physicians’ genders have been found to have a significant impact on primary care physician practice patterns [6]. Female physicians are more likely to work part-time [5], work fewer on-call hours [6, 9], provide less out of office care (e.g., nursing home, home and hospital visits) [6], and take more leaves of absence, including medical or parental leave [9]. Additionally, female primary care physicians tend to see more female patients and fewer geriatric patients compared to their male counterparts [6].

While studies examining primary care practice patterns by gender often point to parental responsibilities as a potential confounding or intervening factor, few have examined parenthood or the interaction between parenthood and gender directly [6]. Most physicians work more than a 40-h week and may experience stress managing professional and personal obligations, including caregiving [10]. Parental leave may also put a financial burden on physician parents. Canadian residents qualify for basic employment insurance leave (a maximum of approximately $550 per week, up to 52 weeks) with some limited top-ups depending on province [11]. Physicians in practice may have even more limited benefits and may be faced with the stress of making arrangements to cover time away [12]. These factors may contribute to the observation that parenthood has a negative impact on career satisfaction and success [5].

Child bearing and child rearing may define and distinguish the career experiences of female compared with male physicians [13], and the impact of parenthood on practice intentions may differ by gender. A prospective study of physicians after graduation showed that practice patterns that have been associated with female physicians (part-time work, more primary care work, less involvement in academic and hospital work) were more common among parents compared to non-parents [5]. A study of the Canadian physician workforce between 1991 and 2006 found having children reduced hours of market work among female physicians while work at home increased twice as much among female physician parents compared to male physician parents [14]. In addition, male physicians' spouses are much less likely to be employed and if employed had fewer hours worked outside the home [14]. Once children were over age 18, differences between male and female primary care physicians’ working hours diminish [15], but over the course of their career, female physicians spend more time on childcare responsibilities [8]. Also, compared to both male parents, and male and female non-parent physicians, female physicians who have children have the lowest self-reported career-success ratings and satisfaction [5].

A better understanding of how the intersection of gender and parenthood shapes primary care practice intentions could inform supports for physician parents. These could include financial support for parental leave [16], resources to identify locums or other forms of coverage for physicians in practice [16], processes to improve reintegration into training and practice following leave, and childcare [17].

Though some existing research examines the feminization of the primary care physician workforce [6] and points to the possibility that parental responsibilities help explain differences in practice between male and female physicians, very little data is available about physicians as parents and, even more rare, medical trainees as parents [18]. This study aims to contribute new information about how having children can affect practice intentions of FM residents, and to explore the interacting effects of parenthood and gender.

## Methods

### Data

We analyzed data from the Family Medicine Longitudinal Survey collected by the College of Family Physicians of Canada from all 17 university-based family medicine residency programs in 2015–2016 and 2016–2017. For both years, surveys were sent to all FM residents within 3 months of program entry and then again within 3 months of program exit. We focus on the exit surveys in the present analysis, and pool the 2016 and 2017 cohorts. We examine responses to questions about practice intentions with respect to comprehensiveness, type, clinical domains, settings, and populations (see full questions in Appendix 1 and Appendix 2).

The survey asks respondents “What is your sex?” and provides the options “Male,” “Female,” and “Prefer not to answer.” While it is plausible that biological differences specific to pregnancy and childbirth may shape intentions, it is likely that socially constructed gender roles play a larger role. It is not possible to distinguish between sex and gender effects in this analysis, so we use the term sex/gender hereafter. Respondents are also asked “Do you have children?” with the options “Yes/Expecting,” “No,” or “Prefer not to answer.” We classify respondents answering “Yes/Expecting” as parents and those selecting “No” as non-parents. Respondents who selected “Prefer not to answer” or with missing responses to either of these two questions were excluded from the analysis.

### Analysis

The survey measured practice intentions on a 5-point Likert scale. Responses were not normally distributed, and we could not assume they could be treated as interval data. Given the number of practice intention variables analyzed, it was impractical to report frequencies across all five categories for each scale. For the purpose of analysis, we dichotomized responses to questions about practice intentions by grouping those selecting “somewhat likely” or “highly likely” and those selecting “neutral,” “somewhat unlikely,” or “highly unlikely.” Dichotomizing responses in this way provides interpretable results where proportions and odds reflect positive intentions for each practice variable. We conducted sensitivity analysis to confirm that results were similar grouping “neutral” with “somewhat likely” or “highly likely.”

We summarized demographic characteristics and the percent of respondents selecting “somewhat likely” or “highly likely” for all survey questions capturing practice intentions. We report results for all respondents and stratified by sex/gender and parenthood (male non-parents, male parents, female non-parents, female parents). We investigated differences by sex/gender and parenthood using chi-square tests. To explore how survey respondents differ from all FM residents, we compared 2017 respondent characteristics with publicly available data from the Canadian Post-M.D. Education Registry (CAPER) [19].

We used logistic regression models and included an interaction between sex/gender and parenthood to examine the relationship between parenthood, sex/gender, and each dichotomized practice intention variable. We estimated adjusted odds ratios using multivariable models with control variables that are associated with parenthood and/or sex/gender and may also shape practice intentions: marital status, location of medical training (Canada or international), age, and childhood geographic environment (inner city/urban/suburban, small town, rural/remote/isolated, mixed (if lived in more than one Appendix 2)). We excluded the number of years in practice since it was collinear with age. We excluded respondents with missing data for practice outcomes from each model. Respondents’ missing data for variables other than sex/gender, parenthood, and the outcome of interest were retained with indicator variables for “missing/prefer not to answer.”

In describing results of logistic regression, we report “odds of intentions for” each practice outcome, as shorthand for odds of selecting “somewhat likely” or “highly likely” vs. selecting “neutral,” “somewhat unlikely,” or “highly unlikely.” We report odds ratios for male parents, female non-parents, and female parents relative to the reference category of male non-parents. We also indicate with an asterisk when the interaction term between gender and parenthood is significant, indicating that the effect of parenthood differs between male and female FM residents (i.e., the ratio of odds ratios defined by (odds among female parents/odds among female non-parents)/(odds among male parents/odds among male non-parents) is significantly different than 1 at *p* < 0.05). We did not examine the interaction between marital status and parenthood, as there were very few unmarried parents within our study population.

Ethics approval for secondary analysis of the FMLS survey data was obtained from the Simon Fraser University Research Ethics Board.

## Results

The percentage of FM residents reporting they are parents or expecting increased from 14.74 to 23.39% between the survey distributed within 3 months of program entry (“entry” questionnaire) and the survey distributed within 3 months of program exit (“exit” questionnaire), indicating that almost 10% of FM residents become parents during residency (Table 1).

**Table 1:** Number (%) of male and female FM residents who are parents and non-parents in program entry and exit questionnaires

	Total	Male non-parent	Male parent	Female non-parent	Female parents	
	*n* (%)	*n* (%)	*n* (%)	*n* (%)	*n* (%)	*p* value (*χ*^2^)
Entry	1 812	532 (79.17)	140 (20.83)	1 013 (88.86)	127 (11.14)	< 0.001
Exit	1 633	441 (71.71)	174 (28.29)	810 (79.57)	208 (20.43)	< 0.001

### Characteristics of parents and non-parents

Two thirds of FM residents exiting programs in 2016 and 2017 are female (Table 2). Almost all male and female parents are married/common law (96.0% and 95.2% respectively). Compared to non-parents, a higher percentage of parents are international medical graduates (IMGs) and lived in non-urban/suburban environments during childhood. A higher percentage of male parents did their residencies in Western Canada compared to other regions, and a higher percentage of female parents did their residencies in Quebec. The percentage of FM residents who are parents increases with age. While 72.4% of male parents exited their residency 2 years after their MD, only 39.9% of female parents did (Table 2).

**Table 2:** Characteristics of FM residents exiting programs in 2016 and 2017, by sex/gender and parenthood (*n*, %)

	Total (*N* = 1 633)	Male non-parent (*N* = 441)	Male parent (*N* = 174)	Female non-parent (*N* = 810)	Female parents (*N* = 208)	
	*n* (%)	*n* (%)	*n* (%)	*n* (%)	*n* (%)	*p* value (*χ*^2^)
Marital status						< 0.001
Single/divorced	687 (42.6)	241 (55.8)	7 (4.1)	429 (53.5)	10 (42.6)	
Married/common law	927 (57.4)	191 (44.2)	166 (96.0)	373 (46.5)	197 (95.2)	
Location of MD training						< 0.001
Canada	1 254 (85.9)	381 (89.0)	125 (74.0)	705 (90.4)	143 (71.9)	
International	222 (14.1)	47 (11.0)	44 (26.0)	75 (9.6)	56 (28.1)	
Region						< 0.001
Ontario	554 (33.8)	133 (30.2)	42 (24.1)	326 (40.3)	51 (24.5)	
Western Canada	642 (39.3)	200 (45.4)	91 (52.3)	269 (33.2)	82 (39.4)	
Atlantic Canada	64 (3.9)	14 (3.2)	9 (5.2)	31 (3.8)	10 (4.8)	
Quebec	375 (23.0)	94 (21.3)	32 (18.4)	184 (22.7)	65 (31.3)	
Age groupings (years)						< 0.001
< 30	910 (58.8)	263 (62.8)	36 (21.3)	572 (74.6)	39 (20.3)	
30–34	420 (27.2)	113 (27.0)	65 (38.5)	167 (21.8)	75 (39.1)	
35+	217 (14.0)	43 (10.3)	68 (40.2)	28 (3.7)	78 (40.6)	
Years since MD awarded						< 0.001
2	1 292 (79.3)	377 (85.7)	126 (72.4)	706 (87.4)	83 (39.9)	
3	137 (8.4)	25 (5.7)	13 (7.5)	40 (5.0)	59 (28.4)	
4+	201 (12.3)	38 (8.6)	35 (20.1)	62 (7.7)	66 (31.7)	
Childhood environment						< 0.001
Inner city/urban/suburban	1 022 (62.9)	297 (68.0)	89 (51.5)	511 (63.2)	125 (60.4)	
Small town	278 (17.1)	70 (16.0)	37 (21.4)	143 (17.7)	28 (13.5)	
Rural/remote/isolated	227 (14.0)	43 (9.8)	28 (16.2)	121 (15.0)	35 (16.9)	
Mixed	98 (6.0)	27 (6.2)	19 (11.0)	33 (4.1)	19 (9.2)	

Across Canada, 2731 FMRs were invited to complete the exit survey over the 2 years analyzed. Response rates were 60.1% (785/1306) in 2016 and 62.8% (895/1425) in 2017. For respondents exiting residency in 2017, the average age was 30.5 years, 61.0% were female, and 14.6% were IMGs. These percentages are comparable to the 1438 family medicine trainees exiting residency in 2017 captured in CAPER data [22] where the average age was 30.1 years, 62.1% were female, and 15.5% were IMGs.

### Intentions for comprehensive care

Two-thirds (66.9%) of FM residents intend to provide comprehensive care to the same group of patients in the first 3 years of practice (Table 3). This percentage is lowest for male non-parents (60.3%) and highest for female parents (77.0%) and male parents (76.3%). We observe higher odds of comprehensive care in the first 3 years of practice among parents in both univariable and multivariable (adjusted) logistic models (Table 4). Confidence in current ability to provide comprehensive care does not differ by parenthood or sex/gender. Female FM residents, and female parents especially, are more likely to express intentions for comprehensive care delivered in one clinical setting. Odds of intention to provide comprehensive care across multiple clinical settings or that care includes a special interest do not differ by sex/gender or parenthood. Female FM residents, and female parents especially, are less likely to report intentions for practices focused on specific clinical areas (Tables 3 and 4).

**Table 3:** Practice intentions among FM residents exiting programs in 2016 and 2017  (*n*, %)

	Total	Male non-parent	Male parent	Female non-parent	Female parent	*p* value (*χ*^2^)
Residents reporting that they are somewhat or highly likely they						
Will commit to providing comprehensive care to the same group of patients in first three years of practice (Q17, *N* = 1 607)	1 075 (66.9)	261 (60.3)	132 (76.3)	528 (65.9)	154 (77.0)	<0.001
Agree with the statement: “I am confident in my current ability to provide comprehensive care to the same group of patients over time.” (Q19, *N* = 1 611)	1 494 (92.7)	399 (92.4)	157 (91.3)	748 (93.2)	190 (93.1)	0.829
Residents reporting that after completing residency, it is somewhat or highly likely they will practice in the following practice types						
Comprehensive care delivered in one clinical setting (e.g., office-based) (Q16a, *N* = 1 612)	990 (61.4)	250 (57.6)	93 (54.1)	500 (62.3)	147 (72.1)	0.001
Comprehensive care provided across multiple clinical settings (e.g., in-hospital, long-term care, office) (Q16b, 1 614)	1 226 (76.0)	328 (75.8)	125 (72.3)	631 (78.5)	142 (69.6)	0.035
Comprehensive care that includes a special interest (e.g., sports medicine, emergency medicine, palliative care, etc.) (Q16c, *N* = 1 614)	1 094 (67.8)	299 (68.9)	118 (68.2)	548 (68.2)	129 (63.6)	0.577
Practice focused on specific clinical areas (e.g., sports medicine, maternity care, emergency medicine, palliative care, hospital medicine) (Q16d, *N* = 1 611)	507 (31.5)	163 (37.6)	48 (27.9)	251 (31.3)	45 (22.1)	0.001
Solo practice (Q15a, *N* = 1 600)	120 (7.5)	38 (8.8)	15 (8.8)	55 (6.9)	12 (5.9)	0.450
Group physician practice (Q15b, *N* = 1 612)	1 520 (94.3)	393 (90.3)	161 (93.6)	772 (96.3)	194 (95.6)	<0.001
Interprofessional team-based practice (Q15c, *N* = 1 609)	1 420 (88.3)	358 (82.5)	142 (83.0)	751 (93.8)	169 (83.3)	<0.001
Practice that includes teaching health profession learners (Q15d, *N* = 1 606)	1 261 (78.5)	335 (77.4)	141 (82.5)	636 (79.6)	149 (73.4)	0.132
Residents reporting it is somewhat or highly likely they will provide care in the following domains in the first 3 years						
Care across the life cycle (Q21a, *N* = 1 612)	1 469 (91.1)	378 (87.1)	154 (89.5)	747 (93.0)	190 (93.6)	0.002
Intrapartum care (Q21b, *N* = 1 608)	606 (37.7)	93 (21.5)	63 (36.6)	349 (43.6)	101 (49.8)	<0.001
Mental health care (Q21c, *N* = 1 608)	1 435 (89.2)	369 (85.4)	150 (87.2)	732 (91.4)	184 (90.6)	0.009
Chronic disease management (Q21d, *N* = 1 605)	1 501 (93.5)	392 (90.7)	157 (91.3)	756 (94.6)	196 (97.0)	0.006
Palliative care/end of life care (Q21e, *N* = 1 607)	1 039 (64.7)	289 (66.7)	124 (72.1)	499 (62.4)	127 (62.9)	0.069
Office-based clinical procedures (Q21f, *N* = 1 603)	1 346 (84.0)	354 (82.1)	145 (85.3)	682 (85.1)	165 (82.1)	0.449
In-hospital clinical procedures (e.g., chest tube insertion, adult lumbar puncture, nasogastric tube insertion) (Q21g, *N* = 1 609)	630 (39.2)	221 (51.2)	80 (46.5)	275 (34.3)	54 (26.6)	<0.001
Residents reporting it is somewhat or highly likely they will provide care in the following practice settings/populations in the first 3 years						
Emergency departments (Q21h, *N* = 1 610)	676 (42.0)	237 (54.7)	94 (54.7)	289 (36.0)	56 (27.6)	<0.001
In-hospital (Q21i, 1 607)	955 (59.4)	279 (64.4)	113 (66.1)	467 (58.4)	96 (47.3)	<0.001
Care in the home (Q21j, *N* = 1 609)	677 (42.1)	182 (42.0)	73 (42.4)	340 (42.5)	82 (40.4)	0.962
Long-term care facilities (Q21k, *N* = 1 609)	660 (41.0)	176 (40.7)	90 (52.3)	314 (39.2)	80 (39.4)	0.015
Marginalized, disadvantaged, and vulnerable populations (Q21l, *N* = 1 606)	849 (52.9)	227 (52.4)	98 (57.3)	420 (52.5)	104 (51.5)	0.662
Rural populations (Q21m, *N* = 1 532)	830 (54.2)	230 (55.8)	104 (61.5)	397 (52.0)	99 (52.9)	0.124
Elderly populations (Q21n, *N* = 1 531)	1 389 (90.7)	370 (89.8)	152 (89.9)	695 (91.1)	172 (92.0)	0.800
First Nations, Inuit, and Métis (Q21o, *N* = 1 610)	671 (41.7)	191 (44.2)	86 (50.0)	317 (39.5)	77 (37.9)	0.032

**Table 4:** Practice intentions among FM residents exiting programs in 2016 and 2017, unadjusted and adjusted odds ratios and 95% confidence intervals for sex/gender and parenthood

FMLS question (question number, response count)	Sex/gender and parenthood	Unadjusted odds	Adjusted odds
	Reference category male, non-parent		
Will commit to providing comprehensive care to the same group of patients in first 3 years of practice (Q17, *N* = 1 607)	Male, parent	*2.12 (1.42–3.16)*	*1.64 (1.03–2.60)*
	Female, non-parent	*1.27 (1.00–1.62)*	1.17 (0.89*–*1.52)
	Female, parent	*2.21 (1.51–3.23)*	*1.77 (1.13–2.77)*
Agree with the statement: “I am confident in my current ability to provide comprehensive care to the same group of patients over time.” (Q19, *N* = 1 611)	Male, parent	0.87 (0.46–1.64)	0.86 (0.42*–*1.79)
	Female, non-parent	1.12 (0.72–1.76)	1.13 (0.71*–*1.79)
	Female, parent	1.12 (0.59–2.15)	1.16 (0.56*–*2.42)
Comprehensive care delivered in one clinical setting (e.g., office-based) (Q16a, *N* = 1 612)	Male, parent	0.87 (0.61–1.24)	0.71 (0.47*–*1.06)
	Female, non-parent	1.21 (0.96–1.55)	*1.34 (1.04–1.71)*
	Female, parent	1.90 (1.32–2.72)*	*1.53 (1.02–2.29)*
Comprehensive care provided across multiple clinical settings (e.g., in-hospital, long-term care, office) (Q16b, 1 614)	Male, parent	0.83 (0.56–1.24)	0.70 (0.45*–*1.10)
	Female, non-parent	1.17 (0.89–1.54)	1.10 (0.83*–*1.47)
	Female, parent	0.73 (0.51–1.06)	0.69 (0.45*–*1.05)
Comprehensive care that includes a special interest (e.g., sports medicine, emergency medicine, palliative care, etc.) (Q16c, *N* = 1 614)	Male, parent	0.97 (0.66–1.42)	0.99 (0.65*–*1.50)
	Female, non-parent	0.97 (0.75–1.24)	0.94 (0.73*–*1.21)
	Female, parent	0.79 (0.55–1.12)	0.82 (0.55*–*1.21)
Practice focused on specific clinical areas (e.g., sports medicine, maternity care, emergency medicine, palliative care, hospital medicine) (Q16d, *N* = 1 611)	Male, parent	*0.64 (0.44–0.94)*	0.72 (0.47*–*1.11)
	Female, non-parent	*0.75 (0.59–0.96)*	*0.72 (0.56–0.92)*
	Female, parent	*0.47 (0.32–0.69)*	*0.52 (0.34–0.80)*
Solo practice (Q15a, *N* = 1 600)	Male, parent	0.99 (0.53–1.85)	0.93 (0.45*–*1.91)
	Female, non-parent	0.77 (0.50–1.18)	0.84 (0.53*–*1.31)
	Female, parent	0.65 (0.33–1.27)	0.60 (0.28*–*1.28)*
Group physician practice (Q15b, *N* = 1 612)	Male, parent	1.56 (0.79–3.11)	1.80 (0.82*–*3.95)
	Female, non-parent	*2.75 (1.69–4.46)*	*2.75 (1.67–4.53)*
	Female, parent	*2.30 (1.10–4.83)*	*2.81 (1.23–6.42)*
Interprofessional team-based practice (Q15c, *N* = 1 609)	Male, parent	1.04 (0.65–1.66)	1.03 (0.60*–*1.77)
	Female, non-parent	*3.19 (2.18–4.66)*	*3.00 (2.03–4.42)*
	Female, parent	1.06 (0.68–1.64)*	1.05 (0.62*–*1.76)*
Practice that includes teaching health profession learners (Q15d, *N* = 1 606)	Male, parent	1.37 (0.87–2.16)	1.42 (0.85*–*2.38)
	Female, non-parent	1.14 (0.86–1.52)	1.27 (0.95*–*1.72)
	Female, parent	0.81 (0.55–1.19)*	0.88 (0.56*–*1.38)*
Care across the life cycle (Q21a, *N* = 1 612)	Male, parent	1.27 (0.72–2.23)	1.38 (0.73*–*2.63)
	Female, non-parent	*1.98 (1.34–2.92)*	*1.95 (1.30–2.91)*
	Female, parent	*2.17 (1.16–4.06)*	*2.59 (1.29–5.19)*
Intrapartum care (Q21b, *N* = 1 608)	Male, parent	*2.11 (1.43–3.10)*	*2.20 (1.43–3.37)*
	Female, non-parent	*2.81 (2.15–3.68)*	*3.05 (2.32–4.02)*
	Female, parent	3.61 (2.52–5.16)*	3.89 (2.60–5.82)*
Mental health care (Q21c, *N* = 1 608)	Male, parent	1.16 (0.69–1.96)	1.32 (0.73*–*2.40)
	Female, non-parent	*1.81 (1.26–2.61)*	*1.85 (1.27–2.70)*
	Female, parent	1.65 (0.96–2.84)	*2.09 (1.13–3.86)*
Chronic disease management (Q21d, *N* = 1 605)	Male, parent	1.07 (0.57–1.99)	1.00 (0.49*–*2.03)
	Female, non-parent	*1.79 (1.15–2.81)*	*1.93 (1.22–3.07)*
	Female, parent	*3.33 (1.39–8.00)*	*3.54 (1.38–9.12)*
Palliative care/end of life care (Q21e, *N* = 1 607)	Male, parent	1.29 (0.87–1.90)	1.17 (0.75*–*1.81)
	Female, non-parent	0.83 (0.65–1.06)	0.87 (0.68*–*1.13)
	Female, parent	0.84 (0.60–1.20)	0.82 (0.54*–*1.22)
Office-based clinical procedures (Q21f, *N* = 1 603)	Male, parent	1.26 (0.77–2.06)	1.13 (0.66*–*1.96)
	Female, non-parent	1.25 (0.91–1.71)	*1.39 (1.01–1.93)*
	Female, parent	1.00 (0.64–1.54)	1.00 (0.61*–*1.64)
In-hospital clinical procedures (e.g., chest tube insertion, adult lumbar puncture, nasogastric tube insertion) (Q21g, *N* = 1 609)	Male, parent	0.83 (0.58–1.18)	0.71 (0.47*–*1.06)
	Female, non-parent	*0.50 (0.39–0.63)*	*0.49 (0.38–0.63)*
	Female, parent	0.35 (0.24–0.50)*	*0.30 (0.20–0.44)*
Emergency departments (Q21h, *N* = 1 610)	Male, parent	1.00 (0.70–1.42)	0.82 (0.55*–*1.23)
	Female, non-parent	*0.47 (0.37–0.59)*	*0.46 (0.36–0.59)*
	Female, parent	*0.32 (0.22–0.45)*	*0.26 (0.17–0.39)*
In-hospital (Q21i, 1 607)	Male, parent	1.08 (0.74 –1.56)	0.98 (0.65*–*1.50)
	Female, non-parent	*0.77 (0.61–0.99)*	*0.76 (0.59–0.98)*
	Female, parent	0.50 (0.35–0.69)*	*0.49 (0.33–0.73)*
Care in the home (Q21j, *N* = 1 609)	Male, parent	1.02 (0.71–1.45)	0.86 (0.57*–*1.29)
	Female, non-parent	1.02 (0.80–1.29)	1.07 (0.84*–*1.37)
	Female, parent	0.93 (0.67–1.31)	0.79 (0.53*–*1.16)
Long-term care facilities (Q21k, *N* = 1 609)	Male, parent	*1.60 (1.12–2.29)*	*1.51 (1.00–2.25)*
	Female, non-parent	0.94 (0.74–1.20)	1.03 (0.80*–*1.32)
	Female, parent	0.95 (0.68–1.34)	0.93 (0.63*–*1.37)*
Marginalized, disadvantaged, and vulnerable populations (Q21l, *N* = 1 606)	Male, parent	1.22 (0.85–1.74)	1.08 (0.71*–*1.63)
	Female, non-parent	1.00 (0.79–1.27)	1.19 (0.92*–*1.52)
	Female, parent	0.96 (0.69–1.35)	0.89 (0.60*–*1.32)
Rural populations (Q21m, *N* = 1 532)	Male, parent	1.27 (0.88–1.83)	0.93 (0.61*–*1.42)
	Female, non-parent	0.86 (0.67–1.09)	0.84 (0.65*–*1.09)
	Female, parent	0.89 (0.63–1.26)	*0.65 (0.43–0.98)*
Elderly populations (Q21n, *N* = 1 531)	Male, parent	1.01 (0.56–1.84)	1.17 (0.58*–*2.37)
	Female, non-parent	1.16 (0.77–1.74)	1.17 (0.75*–*1.81)
	Female, parent	1.30 (0.70–2.41)	1.77 (0.84*–*3.71)
First Nations, Inuit, and Métis (Q21o, *N* = 1 610	Male, parent	1.26 (0.89–1.80)	1.01 (0.66*–*1.54)
	Female, non-parent	0.82 (0.65–1.04)	0.98 (0.75*–*1.27)
	Female, parent	0.77 (0.55–1.08)	0.67 (0.44*–*1.01)

*Indicates the effect of parenthood is significantly different for male and female residents (i.e., the interaction term, or the ratio of odds ratios (female parents/female non-parents)/(male parents/male non-parents) is significantly different from 1 at *p* < 0.05). NOTE: odds ratios significant at p<0.05 are indicated in *italics*

Full regression results, including odds ratios for all control variables, are presented in Appendix 2

### Intentions for practice model

Few FM residents (7.5%) express intentions for solo practice, regardless of sex/gender, and parenthood. Most FM residents (94.3%) express intentions for group physician practice (Table 3), with highest odds among female parents followed by non-parents (Table 4). Female non-parents are most likely to report intentions for interprofessional team-based practice (93.8%), but intentions are high (82.5–93.9%) among all groups (Table 3), a pattern which persists in multivariable odds (Table 4). Intentions for practice that includes teaching do not differ by sex/gender or parenthood (Tables 3 and 4).

### Intentions for practice domains, settings, and populations

Female FM residents, and female parents in particular, were more likely to express intentions for care across the life cycle, mental health care, and chronic disease management. These patterns persist in both unadjusted and adjusted odds ratios. Intentions for intrapartum care were highest among female parents, followed by female non-parents and male parents—all significantly higher than male non-parents. On the other hand, female FM residents and parents especially were less likely to express intentions for in-hospital procedures, other care in hospital, and care in emergency departments. We observed no differences in intentions for care in the home by sex/gender or parenthood. Male parents were more likely to report intentions for care in long-term care facilities. We observed no significant differences in intentions to care for marginalized, rural, elderly, or Indigenous populations, with the exceptions of lower odds of care for rural populations, which emerged as significant for female parents only in multivariable analysis.

### Summary of parenthood, sex/gender, and interaction effects

Parenthood appears particularly relevant for intentions for comprehensive care. Both male and female parents had higher odds of intentions to provide care to the same group of patients within the first years of practice. However, female respondents were more likely to indicate intentions for comprehensive care in one clinical setting (e.g., office-based practice). Both male and female parents and female non-parents had lower odds of intentions for focused practice, though only the gender effect remained significant in multivariable models.

With respect to practice model, patterns appear driven more by gender than parenthood, but differences between female parents and non-parents exist. Odds of intentions for group physician practice were higher among both female parents and non-parents. Odds of intentions for interprofessional team-based practice were higher among female non-parents but not female parents. While odds of intentions for solo practice were low among all groups, a significant interaction term highlights that the impact of parenthood in discouraging solo practice was even greater for female than male FM residents.

With respect to practice domains, settings, and populations, intentions also differ primarily by gender. Adjusted models show higher odds among female FM residents for care across the life cycle, mental health care, and chronic disease management, and lower odds among female FM residents for in-hospital clinical procedures, and practice in emergency departments and hospital. There are, however, notable interaction terms signalling differences between male and female parents, with odds of intrapartum care highest among female parents, odds of rural practice lowest among female parents, and odds of practice in long-term care facilities higher only among male parents.

## Discussion

Almost a quarter of FM residents are parents or became parents during residency. In general, intentions for the provision comprehensive care were higher among parents, and intentions for clinically focused practice lower. Differences in intentions for practice models, domains, and settings/population were driven primarily by gender. In many cases, parenthood has a different effect among female and male FM residents. Even during residency, while three quarters of male parents finish residency in 2 years, fewer than half of female parents do. This is consistent with a previous study that found the effect of having children had twice the impact on female primary care physicians’ working hours as for male primary care physicians [5].

This study contributes new knowledge, building on the existing understanding of the drivers of differences in hours worked and income, and examining the nature of practice within primary care. Though parents and female FM residents have practice intentions that correspond with health system needs for comprehensive care across the life course, supports such as paid parental leave, assistance arranging practice coverage, and childcare may be needed to ensure they can deliver comprehensive care, both during residency and as they transition from residency into practice [16, 17]. The percentage of parents rose from 15% at the beginning of residency to close to 25% at the end of their program. This may simply reflect the age of FM residents, but may also point to the fact that FM residents who become parents during their residency may be able to take parental leave more easily than after they enter practice, as once in practice there is limited financial support to cover their absence and they may need to find locums and pay overhead. Both male and female parents were more likely to take more than 2 years following their MD to complete their residency compared to non-parents, which is consistent with other information about FM residents delaying residency to have a family and taking longer to complete residency while having a family [20].

Our results underscore the need for flexibility in work arrangements, parental leave, and access to childcare to ensure that doctors who are parents can contribute to the primary care workforce. Intentions for office-based and group physician practice models among female parents may reflect the desire to have control over hours worked to protect time for household and caregiving responsibilities [12]. This is consistent with other reports that physicians planning to have families choose to pursue FM compared to hospital specialties due to the flexibility it affords [21] and also that over the course of their career female physician parents spend significantly more time on childcare and other work at home than their male counterparts [8, 14].

### Limitations

Measures of sex and gender, as well as parenthood are limited. The survey asked about the sex of FM residents. While delays in residency completion among female FM residents may to some extent reflect sex differences in leave required for pregnancy, childbirth, and recovery, it is likely socially constructed gender roles that shape longer-term differences in practice intentions. We are not able to distinguish between the effects of sex and gender nor are we able to identify identities outside of the gender binary. We are also missing information about the number or age of children, or intentions for children in the future. While some physicians choose to have their family younger and ramp up their hours when their children are older [22], others may delay parenthood [18] and these individuals cannot be distinguished from physicians who do not intend to become parents within our data. We were unable to link the entry and exit surveys at the individual level, which could have provided insights into change in practice intentions when FM residents become parents.

Our study is preliminary and descriptive. While some significant findings could be spurious, large differences in practice intentions by gender or parenthood signal areas for further exploration by health policy researchers and consideration by health workforce planners. Within the Canadian context, physicians completing family medicine residencies have considerable autonomy in how they choose to structure their practice. Though payment and organizational models differ among provinces, a majority of physicians are in fee-for-service practice and can choose where they practice, what services they offer, and in many cases which patients they take on. This is not unique to Canada, and we expect findings may have relevance in other settings where a range of practice options exist.

## Conclusions

Both parenthood and gender independently shape practice intentions, but in many cases, the effect of parenthood differs for male and female FM residents. Supporting FM residents who are parents may positively impact the availability of comprehensive primary care services, especially since parents are more likely to report intentions for comprehensive practice soon after entering practice.

## Data Availability

Data are available by request from the College of Family Physicians of Canada but are not publicly available.

## Appendix 1

### List of Family Medicine Longitudinal Survey questions describing physician characteristics and practice intentions

5. What is your marital status?

- Single
- Married
- Common-law
- Divorced/Separated
- Widowed
- Prefer not to answer

6. Do you have children?

- Yes/Expecting
- No
- Prefer not to answer

7. What is your sex?

- Female
- Male
- Prefer not to answer

8. Select the ONE statement which best describes the environment in which you grew up PRIOR to university.

- Exclusively/ predominantly inner city
- Exclusively/ predominantly urban/suburban
- Exclusively/ predominantly small town
- Exclusively/ predominantly rural
- Exclusively/ predominantly remote/isolated
- Mixture of environments

9. What year were you awarded your M.D. degree?

…

15. After completing your residency, how likely are you to practice in the following organizational models?

- Solo practice
- Group physician practice
- Interprofessional team-based practice
- Practice that includes teaching health profession learners

16. After completing your residency, how likely are you to practice in the following family medicine practice types?

- Comprehensive care delivered in one clinical setting. (e.g., office –based)
- Comprehensive care provided across multiple clinical settings (in-hospital, long-term care, office).
- Comprehensive care that includes a special interest (such as sports medicine, emergency medicine, palliative care, etc.)
- I plan to focus only on specific clinical areas (such as sports medicine, maternity care, emergency medicine, palliative care, hospital medicine etc.)

17. In your first three years of practice, do you intend to commit to providing comprehensive care to the same group of patients?

18. If very unlikely or somewhat unlikely, what is your primary reason? (check one only)

19. To what extent do you agree or disagree with the following statement: “*I am confident in my current ability to provide comprehensive care to the same group of patients over time.”*

20. How much exposure have you had to the following domains, practice settings, and specific populations in your medical education to date?

- Care across the life cycle
- Intrapartum care
- Mental health care
- Chronic disease management
- Palliative Care/End of life
- Office-based clinical procedures
- In-hospital clinical procedures (e.g. chest tube insertion, adult lumbar puncture, nasogastric tube insertion)
- Practice setting – Emergency departments
- Practice setting – In-hospital
- Practice setting – Care in the home
- Practice setting – Long-term care facilities
- Marginalized, disadvantaged and vulnerable populations
- Rural populations
- Elderly populations
- Aboriginal populations/First Nations, Inuit and Métis

21. In your future practice as a family physician, how likely are you to provide care in each of the following domains, practice settings, and specific populations in the first 3 years?

- Care across the life cycle
- Intrapartum care
- Mental health care
- Chronic disease management
- Palliative Care/End of life
- Office-based clinical procedures
- In-hospital clinical procedures (e.g. chest tube insertion, adult lumbar puncture, nasogastric tube insertion)
- Practice setting – Emergency departments
- Practice setting – In-hospital
- Practice setting – Care in the home
- Practice setting – Long-term care facilities
- Marginalized, disadvantaged and vulnerable populations
- Rural populations
- Elderly populations
- Aboriginal populations/First Nations, Inuit and Métis

22. To what extent do you agree or disagree with the following statement:

*‘I am confident to begin the practice of comprehensive family medicine in any community in Canada.’*

## Appendix 2

**Table 5:** Results of multivariable logistic regression, odds ratios and 95% confidence intervals

	Intend to provide comprehensive care to the same group of patients within first 3 years (Q17)	Confident in ability (Q19)	Comprehensive care - one clinical setting. (e.g. office) (16a)	Comprehensive care - multiple clinical settings (16b)	Comprehensive care that includes a special interest (16c)
*Parenthood* *(reference male, non-parent)*					
Male, parent	*1.64 (1.03-2.60)*	0.86 (0.42-1.79)	0.71 (0.47-1.06)	0.70 (0.45-1.10)	0.99 (0.65-1.50)
Female, non-parent	1.17 (0.89-1.52)	1.13 (0.71-1.79)	*1.34 (1.04-1.71)*	1.10 (0.83-1.47)	0.94 (0.73-1.21)
Female, parent	*1.77 (1.13-2.77)*	1.16 (0.56-2.42)	*1.53 (1.02-2.29)*	0.69 (0.45-1.05)	0.82 (0.55-1.21)
*Marital Status* *(reference single/divorced)*					
Married/Common-law	*1.36 (1.06-1.76)*	1.52 (0.98-2.35)	1.20 (0.95-1.52)	1.15 (0.88-1.51)	0.99 (0.78-1.26)
*Location of MD training* *(reference Canadian MD)*					
IMG	*3.32 (2.22-4.97)*	0.90 (0.50-1.64)	*1.86 (1.29-2.69)*	0.88 (0.61-1.26)	1.10 (0.78-1.55)
*Region* *(reference Ontario)*					
Western Canada	*1.78 (0.13-0.25)*	*1.79 (1.10-2.93)*	1.23 (0.95-1.60)	0.90 (0.66-1.22)	1.20 (0.92-1.57)
Atlantic Canada	*0.07 (0.04-0.12)*	0.51 (0.24-1.09)	0.66 (0.38-1.14)	1.30 (0.62-2.71)	1.53 (0.83-2.82)
Quebec	*0.12 (0.08-0.18)*	1.44 (0.80-2.58)	1.23 (0.90-1.68)	*0.68 (0.48-0.97)*	1.00 (0.73-1.36)
*Age groupings (years)* *(reference <30)*					
30-34	1.03 (0.77-1.36)	0.76 (0.46-1.23)	0.92 (0.71-1.20)	1.03 (0.76-1.40)	0.80 (0.61-1.04)
35+	1.55 (0.98-2.46)	*0.50 (0.26-0.95)*	*1.57 (1.04-2.37)*	0.91 (0.59-1.40)	0.79 (0.53-1.17)
*Childhood environment* *(reference urban/suburban)*					
Small town	1.18 (0.86-1.63)	1.04 (0.61-1.76)	*0.54 (0.41-0.71)*	*1.65 (1.17-2.32)*	1.18 (0.88-1.58)
Rural/remote/isolated	1.37 (0.95-1.96)	0.86 (0.50-1.49)	*0.51 (0.38-0.69)*	*3.42 (2.14-5.46)*	*1.68 (1.20-2.36)*
Mixture of environment	0.96 (0.57-1.63)	1.73 (0.66-4.55)	*0.62 (0.39-0.97)*	1.34 (0.81-2.23)	1.25 (0.78-1.99)
	Focused practice (16d)	Solo practice (15a)	Group physician practice (15b)	Interprofessional team-based practice (15c)	Practice that includes teaching (15d)
*Parenthood* *(reference male, non-parent)*					
Male, parent	0.72 (0.47-1.11)	0.93 (0.45-1.91)	1.80 (0.82-3.95)	1.03 (0.60-1.77)	1.42 (0.85-2.38)
Female, non-parent	*0.72 (0.56-0.92)*	0.84 (0.53-1.31)	*2.75 (1.67-4.53)*	*3.00 (2.03-4.42)*	1.27 (0.95 -1.72)
Female, parent	*0.52 (0.34-0.80)*	0.60 (0.28-1.28)	*2.81 (1.23-6.42)*	1.05 (0.62-1.76)	0.88 (0.56-1.38)
*Marital Status* *(reference single/divorced)*					
Married/Common-law	0.89 (0.70-1.14)	0.73 (0.47-1.13)	1.09 (0.67-1.78)	1.07 (0.73-1.55)	1.07 (0.81-1.42)
*Location of MD training* *(reference Canadian MD)*					
IMG	0.89 (0.61-1.28)	1.46 (0.85-2.50)	1.17 (0.59-2.33)	1.01 (0.63-1.62)	*0.61 (0.41-0.91)*
*Region* *(reference Ontario)*					
Western Canada	*0.56 (0.43-0.74)*	*2.69 (1.51-4.80)*	*0.42 (0.21-0.83)*	*0.38 (0.23-0.61)*	*4.11 (2.97-5.70)*
Atlantic Canada	0.60 (0.33-1.10)	*3.22 (1.22-8.52)*	*0.28 (0.09-0.86)*	0.47 (0.19-1.19)	*2.10 (1.09-4.06)*
Quebec	*0.53 (0.38-0.74)*	*3.13 (1.64-5.97)*	*0.23 (0.11-0.48)*	*0.28 (0.17-0.48)*	*2.34 (1.63-3.37)*
*Age groupings (years)* *(reference <30)*					
30-34	*0.56 (0.43-0.74)*	0.76 (0.46-1.25)	1.10 (0.63-1.91)	0.87 (0.59-1.27)	1.04 (0.74-1.45)
35+	0.89 (0.59-1.36)	1.28 (0.77-2.84)	0.76 (0.36-1.63)	1.24 (0.70-2.19)	0.68 (0.44-1.07)
*Childhood environment* *(reference urban/suburban)*					
Small town	0.98 (0.73-1.32)	1.01 (0.57-1.76)	*0.52 (0.30-0.91)*	*0.64 (0.42-0.96)*	0.98 (0.70-1.37)
Rural/remote/isolated	0.91 (0.66-1.27)	1.65 (0.96-2.83)	0.81 (0.39-1.65)	1.21 (0.70-2.08)	1.36 (0.92-2.02)
Mixture of environment	*1.98 (1.27-3.09)*	1.04 (0.45-2.40)	0.49 (0.22-1.10)	*0.54 (0.30-0.98)*	1.27 (0.74-2.18)
	Care across the life cycle (21a)	Intrapartum care (21b)	Mental health care (21c)	Chronic disease management (21d)	Palliative care/end of life care (21e)
*Parenthood* *(reference male, non-parent)*					
Male, parent	1.38 (0.73-2.63)	*2.20 (1.43-3.37)*	1.32 (0.73-2.40)	1.00 (0.49-2.03)	1.17 (0.75-1.81)
Female, non-parent	*1.95 (1.30-2.91)*	*3.05 (2.32-4.02)*	*1.85 (1.27-2.70)*	*1.93 (1.22-3.07)*	0.87 (0.68-1.13)
Female, parent	*2.59 (1.29-5.19)*	*3.89 (2.60-5.82)*	*2.09 (1.13-3.86)*	*3.54 (1.38-9.12)*	0.82 (0.54-1.22)
*Marital Status* *(reference single/divorced)*					
Married/Common-law	1.13 (0.76-1.67)	0.87 (0.69-1.11)	1.11 (0.77-1.60)	1.04 (0.67-1.64)	1.02 (0.80-1.30)
*Location of MD training* *(reference Canadian MD)*					
IMG	1.73 (0.93-3.20)	0.94 (0.68-1.32)	0.85 (0.52-1.41)	1.72 (0.77-3.83)	0.96 (0.67-1.36)
*Region* *(reference Ontario)*					
Western Canada	0.81 (0.51-1.30)	*1.96 (1.50-2.57)*	1.41 (0.93-2.14)	1.22 (0.72-2.08)	*2.73 (2.08-3.57)*
Atlantic Canada	*0.35 (0.16-0.77)*	*1.74 (1.00-3.05)*	1.42 (0.54-3.76)	0.65 (0.24-1.78)	*2.46 (1.33-4.53)*
Quebec	0.76 (0.44-1.34)	*1.49 (1.08-2.05)*	1.13 (0.68-1.85)	0.97 (0.52-1.82)	*2.27 (1.64-3.13)*
*Age groupings (years)* *(reference <30)*					
30-34	0.99 (0.62-1.57)	0.88 (0.67-1.16)	0.87 (0.57-1.33)	1.13 (0.66-1.92)	1.05 (0.79-1.39)
35+	0.54 (0.30-1.00)	1.01 (0.68-1.50)	0.60 (0.34-1.05)	0.97 (0.46-2.07)	0.89 (0.59-1.33)
*Childhood environment* *(reference urban/suburban)*					
Small town	1.15 (0.69-1.91)	0.90 (0.67-1.21)	1.06 (0.66-1.68)	1.05 (0.59-1.88)	*1.58 (1.17-2.14)*
Rural/remote/isolated	0.99 (0.58-1.72)	1.31 (0.96-1.78)	1.00 (0.60-1.67)	0.90 (0.49-1.68)	*1.91 (1.36-2.69)*
Mixture of environment	0.67 (0.34-1.31)	1.20 (0.77-1.89)	0.75 (0.41-1.40)	0.61 (0.28-1.32)	1.13 (0.71-1.79)
	Office-based clinical procedures (21f)	In-hospital clinical procedures (21g)	Emergency departments (21h)	In-hospital (21i)	Care in the home (21j)
*Parenthood* *(reference male, non-parent)*					
Male, parent	1.13 (0.66-1.96)	0.71 (0.47-1.06)	0.82 (0.55-1.23)	0.98 (0.65-1.50)	0.86 (0.57-1.29)
Female, non-parent	*1.39 (1.01-1.93)*	*0.49 (0.38-0.63)*	*0.46 (0.36-0.59)*	*0.76 (0.59-0.98)*	1.07 (0.84-1.37)
Female, parent	1.00 (0.61-1.64)	*0.30 (0.20-0.44)*	*0.26 (0.17-0.39)*	*0.49 (0.33-0.73)*	0.79 (0.53-1.16)
*Marital Status* *(reference single/divorced)*					
Married/Common-law	0.88 (0.65-1.20)	0.92 (0.73-1.17)	0.90 (0.71-1.14)	0.87 (0.69-1.10)	0.91 (0.72-1.15)
*Location of MD training* *(reference Canadian MD)*					
IMG	1.46 (0.88-2.43)	0.74 (0.52-1.05)	0.87 (0.62-1.22)	0.99 (0.71-1.39)	*1.51 (1.09-2.10)*
*Region* *(reference Ontario)*					
Western Canada	*2.50 (1.75-3.58)*	1.76 (1.35-2.30)	*2.09 (1.60-2.73)*	1.22 (0.94-1.59)	*1.98 (1.52-2.58)*
Atlantic Canada	0.94 (0.48-1.84)	1.17 (0.66-2.09)	*1.84 (1.05-3.24)*	1.72 (0.93-3.19)	*2.40 (1.39-4.15)*
Quebec	1.44 (0.97-2.14)	1.33 (0.96-1.83)	*1.52 (1.10-2.11)*	0.76 (0.56-1.03)	*2.92 (2.13-3.99)*
*Age groupings (years)* *(reference <30)*					
30-34	1.17 (0.81-1.69)	*1.36 (1.04-1.77)*	*1.31 (1.01-1.71)*	1.27 (0.97-1.67)	1.03 (0.80-1.35)
35+	1.02 (0.61-1.71)	1.08 (0.72-1.61)	1.15 (0.77-1.71)	1.03 (0.69-1.52)	1.17 (0.80-1.35)
*Childhood environment* *(reference urban/suburban)*					
Small town	*1.54 (1.03-2.30)*	*1.64 (1.24-2.19)*	*1.97 (1.48-2.62)*	*1.52 (1.14-2.02)*	*1.44 (1.09-1.91)*
Rural/remote/isolated	1.30 (0.85-1.99)	*3.07 (2.25-4.19)*	*2.83 (2.06-3.87)*	*2.55 (1.81-3.60)*	*1.83 (1.35-2.49)*
Mixture of environment	1.01 (0.57-1.79)	*2.36 (1.50-3.69)*	*2.14 (1.36-3.38)*	0.96 (0.61-1.50)	1.12 (0.72-1.76)
	Long-term care facilities (21k)	Marginalized, disadvantaged and vulnerable (21l)	Rural populations (21m)	Elderly populations (21n)	First Nations (21o)
*Parenthood* *(reference male, non-parent)*					
Male, parent	*1.51 (1.00-2.25)*	1.08 (0.71-1.63)	0.93 (0.61-1.42)	1.17 (0.58-2.37)	1.01 (0.66-1.54)
Female, non-parent	1.03 (0.80-1.32)	1.19 (0.92-1.52)	0.84 (0.65-1.09)	1.17 (0.75-1.81)	0.98 (0.75-1.27)
Female, parent	0.93 (0.63-1.37)	0.89 (0.60-1.32)	*0.65 (0.43-0.98)*	1.77 (0.84-3.71)	0.67 (0.44-1.01)
*Marital Status* *(reference single/divorced)*					
Married/Common-law	0.81 (0.64-1.02)	0.89 (0.70-1.13)	1.06 (0.83-1.34)	1.25 (0.82-1.90)	1.08 (0.84-1.38)
*Location of MD training* *(reference Canadian MD)*					
IMG	*1.27 (1.06-2.03)*	1.10 (0.78-1.55)	1.25 (0.88-1.77)	1.51 (0.79-2.90)	0.79 (0.56-1.11)
*Region* *(reference Ontario)*					
Western Canada	*2.72 (2.09-3.54)*	*4.34 (3.33-5.67)*	*1.54 (1.19-2.00)*	1.30 (0.82-2.07)	*8.09 (6.02-10.86)*
Atlantic Canada	1.59 (0.91-2.78)	*3.55 (2.02-6.24)*	*2.41 (1.32-4.42)*	1.42 (0.48-4.20)	*1.98 (1.10-3.56)*
Quebec	*1.72 (1.25-2.36)*	*2.43 (1.78-3.31)*	*1.75 (1.28-2.41)*	1.30 (0.72-2.35)	*2.70 (1.92-3.80)*
*Age groupings (years)* *(reference <30)*					
30-34	0.91 (0.70-1.18)	1.22 (0.93-1.59)	*1.33 (1.01-1.75)*	0.74 (0.46-1.19)	1.13 (0.86-1.49)
35+	1.16 (0.79-1.70)	1.14 (0.77-1.70)	1.15 (0.77-1.72)	*0.42 (0.22-0.79)*	1.10 (0.73-1.65)
*Childhood environment* *(reference urban/suburban)*					
Small town	1.26 (0.95-1.68)	0.98 (0.73-1.30)	2.14 (1.60-2.86)	1.22 (0.70-2.12)	1.22 (0.90-1.65)
Rural/remote/isolated	*1.54 (1.13-2.10)*	1.30 (0.94-1.78)	*5.08 (3.53-7.31)*	1.03 (0.58-1.83)	*1.98 (1.42-2.76)*
Mixture of environment	1.03 (0.66-1.62)	0.79 (0.50-1.25)	*1.81 (1.14-2.87)*	0.68 (0.34-1.35)	1.57 (0.97-2.53)

Note: odds ratios significant at *p* <0.05 are indicated in *italics*

## Abbreviations

CAPER: Canadian Post-M.D. Education Registry
CaRMS: Canadian Residency Matching Service
FM: Family medicine
IMGs: International medical graduates
